# Comparison of autologous platelet concentrates and topical steroids on oral lichen planus: a systematic review and meta-analysis

**DOI:** 10.1186/s12903-024-04443-y

**Published:** 2024-06-08

**Authors:** Bita Azizi, Katayoun Katebi, Hosein Azizi, Maryam Hosseinpour Sarmadi

**Affiliations:** 1 Department of Oral and Maxillofacial Medicine, Faculty of Dentistry, Tabriz University of Medical Sciences, Tabriz, Iran; 2 Women’s Reproductive Health Research Center, Tabriz University of Medical Sciences, Tabriz, Iran

**Keywords:** Lichen planus, oral, Platelet-rich plasma, Platelet-rich fibrin

## Abstract

**Background:**

Oral lichen planus is a chronic and potentially malignant disorder of oral mucosa. Corticosteroids are used as first-line therapy for oral lichen planus patients; however, they have many side effects. Platelet concentrates (platelet-rich plasma and platelet-rich fibrin) are autologous bioactive materials. This systematic review investigated the effects of autologous platelet concentrates compared to topical steroids in treating symptomatic oral lichen planus patients.

**Materials and methods:**

A systematic literature search was performed in PubMed, Web of Science, Scopus, Embase, and Cochrane for randomized controlled trials. Preferred Reporting Items for Systematic Reviews and meta-analysis guidelines were observed for article selection. For the pooling of studies, meta-analysis using Standardized Mean Differences by random effects model was carried out to estimate summary effect sizes for the treatment of oral lichen planus.

**Results:**

A total of six studies, incorporating 109 oral lichen planus patients, were involved. Both treatment modalities showed a statistically significant improvement in the outcome parameters (lesion size, pain score, Thongprasom score) from the baseline to the end of treatment and further to the follow-up visits. There was no significant difference in the pooled estimate SMD of pain decline in patients of the two groups (SMD = 0.17 (95% CI: -0.47 to 0.81); I^2^ = 63.6%). The SMD of Thongprasom score in patients receiving autologous platelet concentrates was lower than the corticosteroid groups, with no significant effect size (SMD= -2.88 (95% CI: -5.51 to -0.25); I^2^ = 91.7%). Therefore, there was no statistically significant difference between the autologous platelet concentrates and topical steroids regarding pain and clinical score.

**Conclusion:**

Autologous platelet concentrates, and topical steroids decreased the size of lesions, Thongprasom scale, and pain in oral lichen planus patients, but the difference between the two treatments was not statistically significant. Thus, autologous platelet concentrates could be considered as an alternative treatment to topical steroids.

**Supplementary Information:**

The online version contains supplementary material available at 10.1186/s12903-024-04443-y.

## Introduction

Lichen planus is a chronic autoimmune mucocutaneous condition [1] that involves oral and genital mucous membranes, skin, nails, and scalp [2]. Its prevalence is about 5% in the general adult population and has a female predilection of 2 to 1 [3]. Approximately 77% of patients with lichen planus show oral manifestations [4]. Oral lichen planus (OLP) is a chronic disorder with a global prevalence of 0.1 to 3.2% [5]. It usually appears in 50 to 70-year-old women [6]. 

The etiology of OLP is unknown, but it is considered a multifactorial process; psychological problems, infections, malnutrition, allergy, endocrine disorders, and genetic susceptibility have been reported as possible triggering factors [7]. The development of a chronic, dysregulated immune response to OLP-mediating antigens leads to increased cytokine, chemokine, and expression of adhesion molecules, which results in keratinocyte cell death, mucosal basement membrane destruction, and long-term chronicity of the disease [8]. This immune response is presumed to be mediated by CD4 + and CD8 + T-lymphocytes [9]. Oral lichen planus is characterized by white striae, known as Wickham’s striae, which highly indicate OLP. It can be reticular, popular, plaque-like, erosive (ulcerated), atrophic, and bullous. Atrophic, erosive, and bullous forms are associated with symptoms such as burning sensations and pain [9]. 

For many patients, OLP considerably limits their essential daily activities, such as eating, drinking, talking, or interacting with others [10]. Despite being a benign disorder,1.4% of oral lesions transform into malignancy, and the World Health Organization has categorized OLP as an oral potentially malignant disorder (OPMD) [11]. Ulceration, location on the tongue, and female sex are reported as possible risk factors for malignant transformation [12]. A recent systematic review on this subject concluded that OLP behaves as an OPMD, whose malignancy ratio is probably underestimated due to inadequate diagnostic criteria and the low methodological quality of the studies [13]. 

Currently, the treatment of OLP focuses on reducing ulcerations and symptoms and possibly increasing the disease-free period. Corticosteroids (CSs), calcineurin inhibitors, retinoids, photodynamic therapy, and natural alternatives are current treatment options; however, their efficacy degrees vary [14, 15]. Corticosteroids can be administered as first-line therapy by topical, intralesional, or systemic routes. Topical use of CSs poses a risk of oral candidiasis and tachyphylaxis. During long-term treatment courses with systemic CS, the patient becomes susceptible to Cushing’s syndrome, hypertension, diabetes, gastric ulcers, and immune suppression. Thus, an effective treatment method with fewer or no side effects is needed.

Autologous platelet concentrates (APCs, including platelet-rich plasma and platelet-rich fibrin) are autologous bioactive materials with various applications in the medical and dental fields. The foundation of these preparations is to extract specific elements from the patient’s blood and use them for indorsing tissue regeneration. First-generation platelet concentrate, called platelet-rich plasma (PRP), contains high concentrations of platelets but negligible natural fibrinogen. Platelet-rich fibrin (PRF) is a second-generation platelet concentrate that accelerates soft and hard tissue healing. Its ease of preparation and application, lower cost, and lack of need for biochemical modification give it an advantage over PRP [16]. 

These products have higher growth factors than the usual amounts necessary for regeneration and tissue repair [17]. Platelet-derived growth factors (PGFs) are important in inflammation, proliferation, and remodeling, the three phases of wound healing and repair cascade. Activated platelets release several growth factors leading to cell proliferation, differentiation, neo-angiogenesis, toxins removal, and cell regeneration. No side effects have been reported with autologous platelet concentrates [18]. 

Considering that several studies have investigated the effects of APCs on oral lichen planus compared to topical steroids, we have done this systematic review and meta-analysis to compare the summary effects of APCs on treating oral lichen planus with topical steroids.

## Methods

This systematic review study is done following the Preferred Reporting Items for Systematic Reviews and Meta-Analyses (PRISMA) [19]. The study protocol has been registered in PROSPERO (Registration ID: CRD42022329977). The principal question of this study was formulated based on the “PICO” (population, intervention, comparison, and outcome) approach, where “P” indicates patients diagnosed with oral lichen planus who need treatment, “I” indicates Autologous Platelet Concentrates, including platelet-rich fibrin and platelet-rich plasma, “C” indicates topical steroids and “O” indicates changes in the pain based on visual analog scale (VAS) or numerical rating scale (NRS), changes in the clinical presentations based on Thongprasom scale, and changes in the lesion size. Therefore, the research question was,” Are there any differences regarding pain and clinical presentations between Autologous Platelet Concentrates and topical steroids in the treatment of oral lichen planus?”.

### Search strategy

Electronic research without restriction on publication start date was carried out until 30 December 2023 using five primary electronic databases: PubMed, the Cochrane Central Register for Controlled Trials, Web of Science, Scopus, and Embase.

Every possible combination of free and MESH (Medical Subject Heading) terms with “OR” and “AND” operators was used for searching. The reference lists of the included articles were also searched to identify more research studies. The search keywords were “oral lichen planus”, “oral lichenoid reactions”, “oral lichenoid lesions”, “platelet-rich-plasma”, “platelet-rich-fibrin”, “platelet-rich fibrin”, “platelet-rich plasma”, “thrombocyte rich fibrin”, “thrombocyte rich plasma”.

The EndNote Basic software was used to manage the references, and duplicate references were identified and removed. The exact search keywords are provided in Appendix 1.

### Eligibility criteria

Studies were included if they were randomized, controlled clinical trials, and published in English. Studies were excluded if they were semi-experimental studies, In-vitro or animal studies, Reprinted articles that use information from the same sample, Letters to the editor and correspondence, Review articles, and Studies with limited information that do not provide the absolute frequency of outcomes and independent variables.

### Screening and selection

Two independent reviewers (K.K. and B.A.) screened the titles. In the next stage, the abstracts were analyzed to ensure their compliance with the eligibility criteria. The full texts of the remaining articles were reviewed to select the final articles that met the inclusion criteria. The authors discussed with the third reviewer (M.H.S.) whenever there was any disagreement. Cohen’s Kappa score was used to assess the level of agreement between the reviewers.

### Data extraction

After the final selection of studies, the required information was extracted and summarized using a table designed in the Microsoft Excel software environment. First author, year, country, study duration in months, follow-up in months, mean age, gender of participants, total sample size, size of lesions, VAS score, and Thongprasom score were extracted from the included studies by two independent reviewers (K.K. and B.A.).

### Risk of bias assessment

The revised Cochrane risk-of-bias tool for randomized trials (RoB2) [20] was used by two independent reviewers (M.H.S and B.A) to assess the risk of bias. Disagreements were discussed with a third reviewer (K.K.). RoB2 is structured in five domains and a judgment of the overall risk of bias.

### Outcome parameters

The outcomes of this article based on PICO were changes in the size of the lesions in mm^2^, changes in the pain and burning sensation evaluated by visual analogue scale (VAS) or numeric rating scale (NRS), and changes in the clinical score.

The visual analogue scale (VAS) and numeric rating scale (NRS) are validated measurements for acute and chronic pain [21]. NRS and VAS are not identical scales; however, they have similarities [22], so they can be compared to each other in a meta-analysis using the standard method.

VAS scores are recorded by making a handwritten mark on a 10-cm line representing a continuum between “no pain = 0” and “worst pain = 10”. The patient rates the current pain level by placing a mark on the line [21]. 

NRS is an 11-point scale, on which 0 represents ‘‘no pain’’ and 10 represents either ‘‘the worst possible pain’’ or ‘‘the most intense pain imaginable’’ [23]. 

Thongprasom score is used for clinical evaluation of the size and shape of oral lichen planus lesions, which varies from 0 to 5: score 0, normal mucosa; score 1, a lesion having only white striae; score 2, a lesion with white striae and atrophic areas less than 1 cm^2^; score 3, a lesion with white striae and atrophic areas larger than 1 cm^2^; score 4, a lesion with white striae and erosive areas less than 1 cm^2^; and score 5, a lesion with white striae with erosive regions larger than 1 cm^2^ [24]. 

### Statistical analysis

The Standardized Mean Differences (SMD), endpoint scores, or change scores were used as effect sizes since the studies had different measuring scales (NRS and VAS). The values were compared between intervention and control groups. SMD has calculated the difference of values between intervention and control groups divided into pooled Standard Deviation (SD). Pooled SMDs and 95% CIs were calculated using the Der Simonian and Laird method via the random effects model. Cochran’s Q test and I^2^ were measured to assess the heterogeneity between studies [25]. All statistical analyses were performed by STATA 14.0 (StataCorp, College Station, TX, US).

## Results

**Fig. 1:**
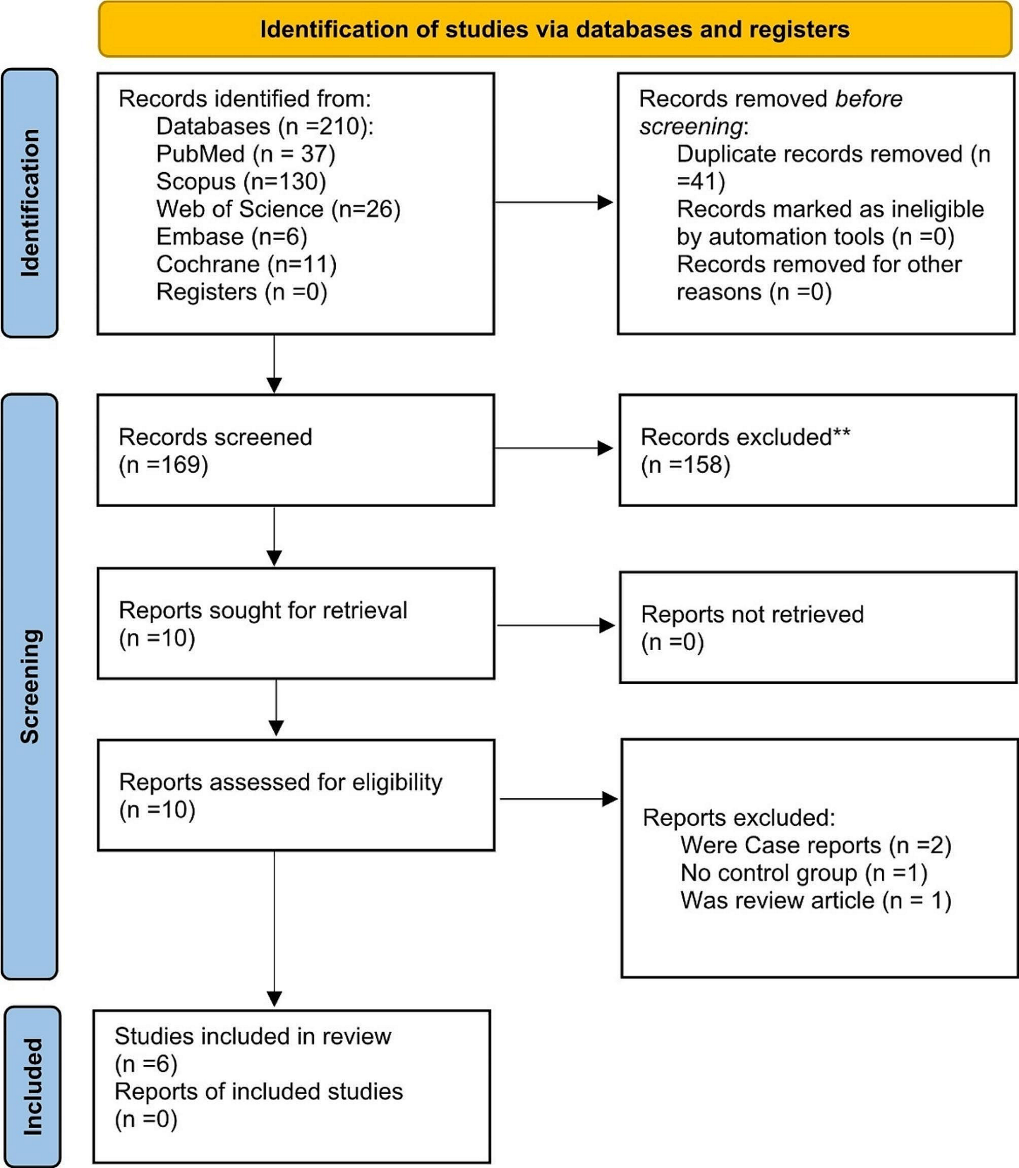
PRISMA flowchart of the articles’ selection process

The electronic search in the mentioned databases yielded 210 articles. After removing the duplicates, 169 articles were screened; out of the 169 articles, 10 were related to the subject, from which two were case reports, and one was a review. Seven articles entered the full-text stage, but one did not have a control group; at last, six articles fulfilled the inclusion criteria. The details of the search results are presented in the PRISMA 2020 flow diagram (Fig. 1). The k value for inter-reviewer agreement for article selection for both abstract and full-text article steps was 0.87, indicating an “almost perfect” agreement.

### Characteristics of the studies

The descriptive characteristics of the included studies are presented in Table 1. The total number of participants in these six studies was 109. The publication date of the studies ranged from 2020 to 2023. All of the six studies were randomized controlled trials (RCT). Three studies were split-mouth designed. The majority of the patients included in the studies were females (79 out of 109 patients; 72.5%). The mean age of the patients ranged from 42.6 to 59.5.

**Table 1:** The characteristics of the studies included in the review

First author, year	Country	Female/Male	Mean age	Sample size		OLP type	Therapy Duration (months)	Follow up (months)
Ahuja [26] 2020	India	18/2	44.5	20	Intervention (10)	Erosive	2	4
					Control (10)			
Hijazi [8] 2021	Egypt	18/2	Intervention:42.6	20	Intervention (10)	Erosive	1	3
			Control:50.3		Control (10)			
Saglam [31] 2021	Turkey	14/10	Intervention:52.2	24	Intervention (24)	Erosive	2	6
			Control:52.2		Control (24)			
Bennardo [30] 2020	Italy	6/3	Intervention:59.5	9	Intervention (9)	NR	2	5–9
			Control:59.5		Control (9)			
Al-Hallak [29] 2022	Syria	9/3	Intervention:48	12	Intervention (12)	plaque-like, ulcerative, atrophic, erosive	1	3
			Control:48		Control (12)			
El Ghareeb [27] 2023	Egypt	14/10	Intervention:47	24	Intervention (12)	Erosive, reticular, mixed	2	3
			Control:52.17		Control (12)			

NR: Not Reported; OLP: Oral Lichen Planus

**Table 2:** The results of the studies included in the review

First author, year	Treatment modalities		Lesion Size in mm^2^		Pain (VAS, NRS)		Thongprasom score		
			Before	After	Before	After	Before	After	
Ahuja [26] 2020	Intervention	Intralesional injections of PRP	460 ± 96.6	60 ± 96.6	8.90 ± 0.99	0.6 ± 0.9	2.6 ± 0.4	0.3 ± 0.4	
	Control	Intralesional injections of TA	465 ± 62.5	110 ± 119.7	8.7 ± 0.9	1.6 ± 2.2	2.7 ± 0.4	0.9 ± 1.1	
Hijazi [28] 2021	Intervention	Intralesional injections of PRP	NR	NR	6.9	2	4.4	1.9	
	Control	Intralesional injections of TA	NR	NR	8.5	1.7	4.1	1.9	
Saglam [312] 2021	Intervention	Intralesional injections of PRF	NR	NR	8.1 ± 1.7	1.3 ± 1.8	4.7 ± 0.4	1.8 ± 1.0	
	Control	Intralesional injections of methylprednisolone acetate	NR	NR	8.0 ± 1.7	2.3 ± 2.6	4.7 ± 0.4	2.2 ± 1.3	
Bennardo [30] 2020	Intervention	Intralesional injections of PRF	318.7 ± 121.1	127.6 ± 59.4	5.9 ± 2	2.9 ± 2.1	2.5 ± 0.6	2.2 ± 0.6	
	Control	Intralesional injections of TA	292.8 ± 119	137.4 ± 78.8	4.6 ± 2.5	1.9 ± 1.5	2.5 ± 0.6	2.2 ± 0.8	
Al-Hallak [29] 2022	Intervention	Intralesional injections of PRF	NR	NR	6.0 ± 2.1	1.9 ± 1.3	NR	NR	
	Control	Intralesional injections of TA	NR	NR	6.3 ± 2.2	0.5 ± 0.7	NR	NR	
El Ghareeb [27] 2023	Intervention	Intralesional injections of TA	NR	NR	5.8 ± 2.4	3 ± 2.6	NR	NR	
	Control	Intralesional injections of PRP	NR	NR	6.2 ± 2.3	2.5 ± 3.4	NR	NR	

NR: not reported; TA: triamcinolone acetonide; VAS: visual analogue scale; NRS: numeric rating scale

Five studies used VAS as a pain assessment scale before and after the interventions, and one used NRS as a pain assessment tool. Four out of six studies used the Thongprasom scale as a clinical score before and after treatment for both the intervention and control groups. Two studies compared the lesion size in mm^2^ before and after interventions.

Three studies used PRP, and three studies used PRF as platelet concentrate. The applied corticosteroid in the studies was triamcinolone acetonide (TA) in five studies and methylprednisolone acetate in one study. Both treatment modalities were applied as injections in all studies (Table 2).

In the study of Ahuja et al., one group of patients was given bilateral intralesional injections with 10 mg/ml of triamcinolone acetonide (TA), and another group was given bilateral intralesional injections of autologous PRP. The injections were given weekly for eight weeks. The injections in both groups were given after a field block local anesthetic with a vasoconstrictor. 0.5 ml of either corticosteroid or PRP was injected per 1cm^2^ of the involved mucosa. Significant reduction in the mean pain scores and the mean lesion size was observed in both groups, but the comparative *p* values were found to be insignificant [26]. 

In the study conducted by El Ghareeb et al., PRP Injections were given at four points of the lesion’s periphery (superior, inferior, left, and right) in one group, and intralesional injection of triamcinolone acetonide as multiple 0.2-ml injections at 1-cm intervals in the other group. 40 mg/ml of TA was mixed with 1 ml of lidocaine 2%, and the final concentration of TA was 20 mg/ml. The injection was performed for both groups every two weeks for two months. There were no statistically significant differences between the studied groups in pain score (NRS) after treatment [27]. 

In the study conducted by Hijazi et al., two groups of patients received intralesional injections of either PRP or 40 mg/ml of TA. 0.5 ml of each treatment was injected per 1 cm^2^ of the ulcerated mucosa. The injections in both groups were applied after a field block with Mepivacaine 3% anesthetic without vasoconstrictor. The patients in both groups received injections once a week for four weeks. There was no statistical significance when comparing the two groups regarding pain and clinical score or remission [28]. 

In the split-mouth study conducted by Al-Hallak et al., patients received an intralesional injection of 1 ml of PRF on one side and an intralesional injection of 0.5 ml of triamcinolone acetonide (40 mg/ml) on the other side. The control side (TA) injections were done 15 days after finishing the treatment of the study side (PRF). Both treatments were applied once a week for four weeks. There was no significant difference between the groups regarding the pain score [29]. 

In the split-mouth study conducted by Bennardo et al., the test side received 1 mL of PRF injection, and the control side received 0.5 ml of triamcinolone acetonide (40 mg/ml). The treatments were applied once a week for a month. For each patient, experimentation lasted eight weeks. Both treatments effectively reduced the lesions’ extension and improved symptoms. However, no statistically significant difference was observed comparing changes in lesion extension and pain modification between the two protocols [30]. 

In the split-mouth study by Saglam et al., one side received 40 mg/ml of methylprednisolone acetate injections, and the other side received PRP injections. Methylprednisolone acetate was injected at four different points into the subepithelial tissue underlying the lesion and adjacent to the normal mucosa. Each injection was 0.2 mL per session. PRF was injected at four different points at the periphery of the lesion. The treatments were applied in four sessions at 15-day intervals. The intergroup comparison showed no significant difference between the PRF and corticosteroid groups regarding VAS-pain values and Thongprasom score [31]. 

### Assessing the risk of bias

**Fig. 2:**
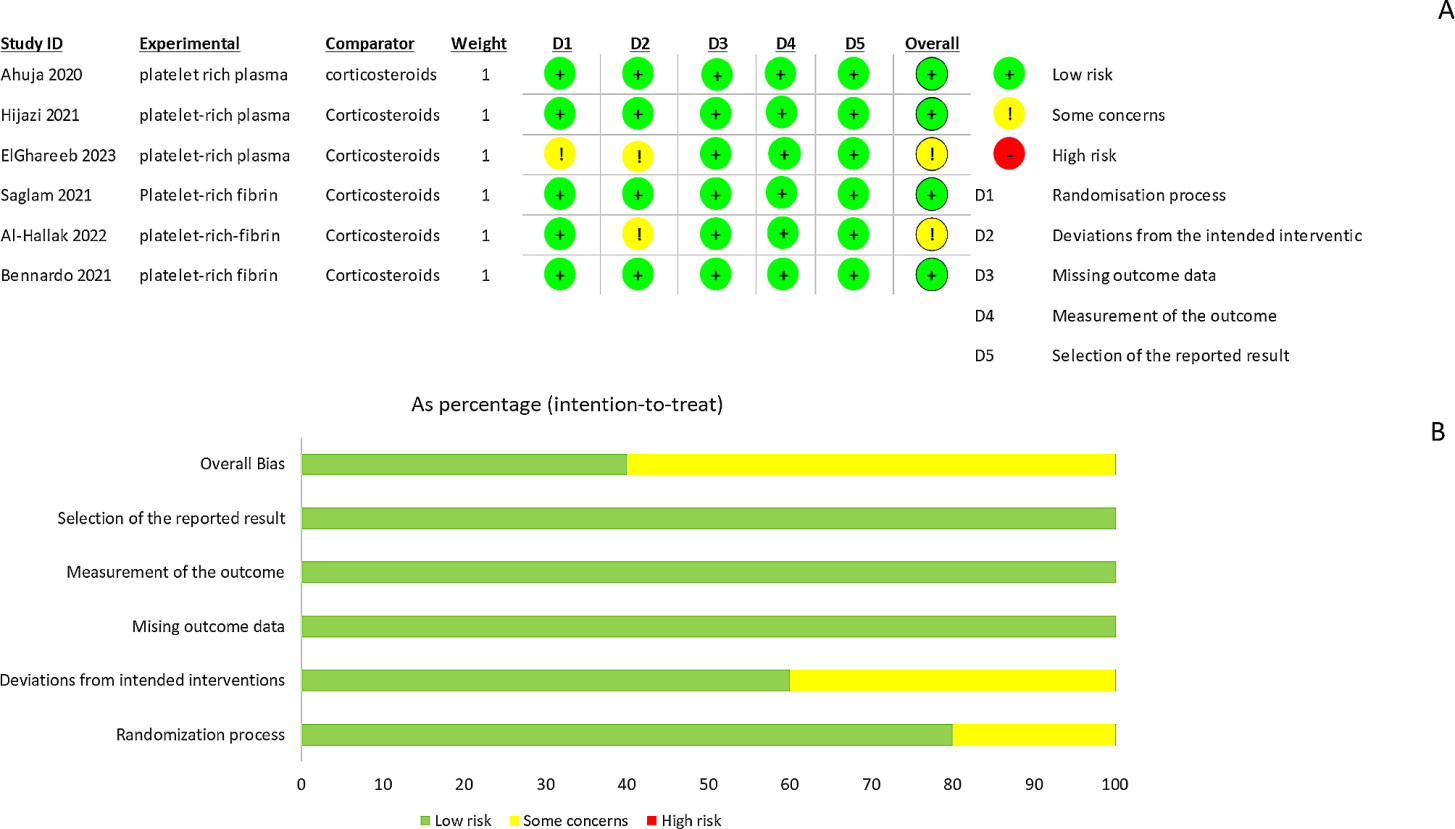
(**A**) The risk of bias for each study; (**B**) Risk of bias in each domain, based on Cochrane risk of bias tool 2

According to the RoB2 tool, out of six RCT studies, four showed a low risk of bias, whereas the other two showed some concerns (Fig. 2). Randomization of the samples wasn’t clearly indicated in one study (26), and two studies didn’t mention the blindness of the assessor [27, 29].

### Meta-analysis

**Fig. 3:**
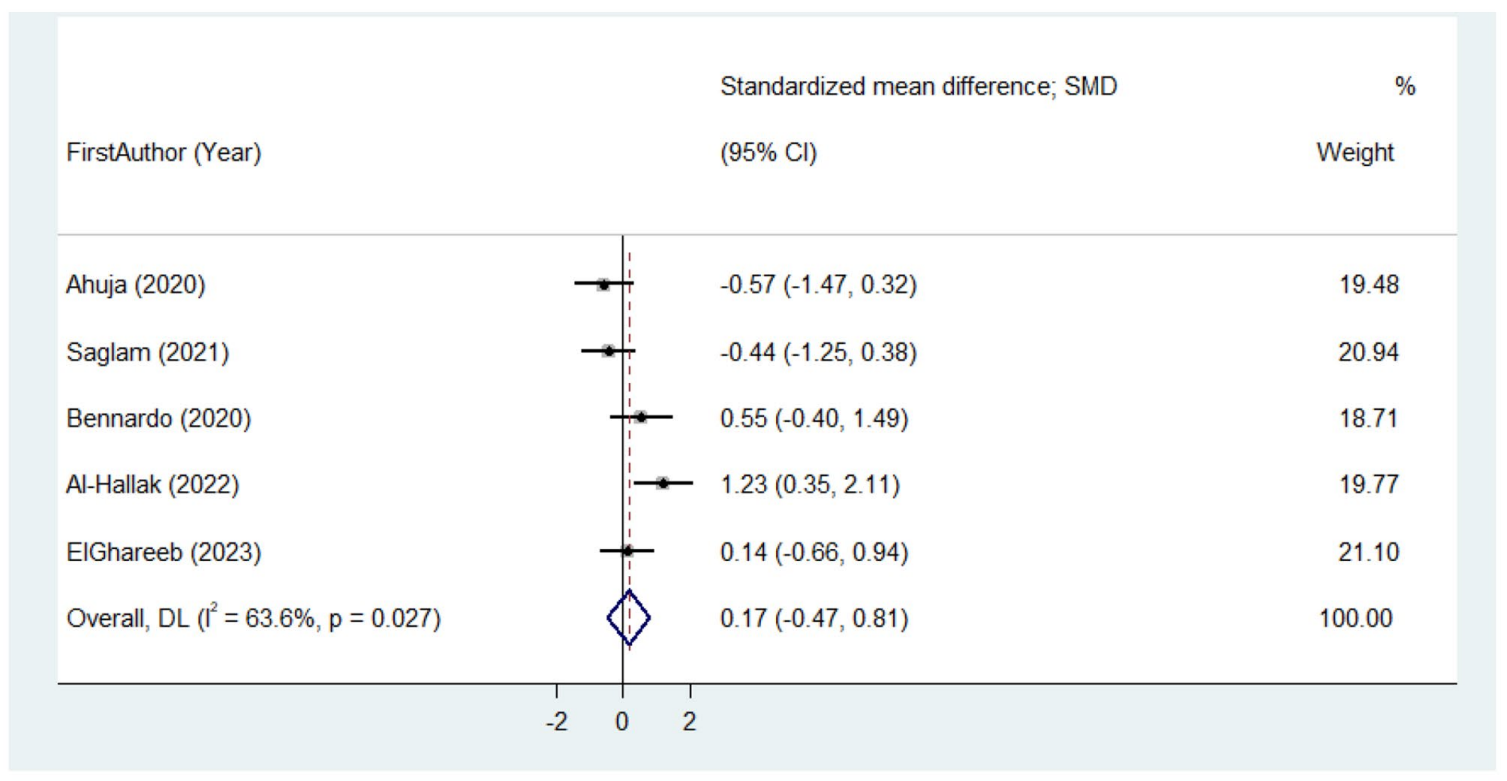
Meta-analysis of standardized mean difference of pain

**Fig. 4:**
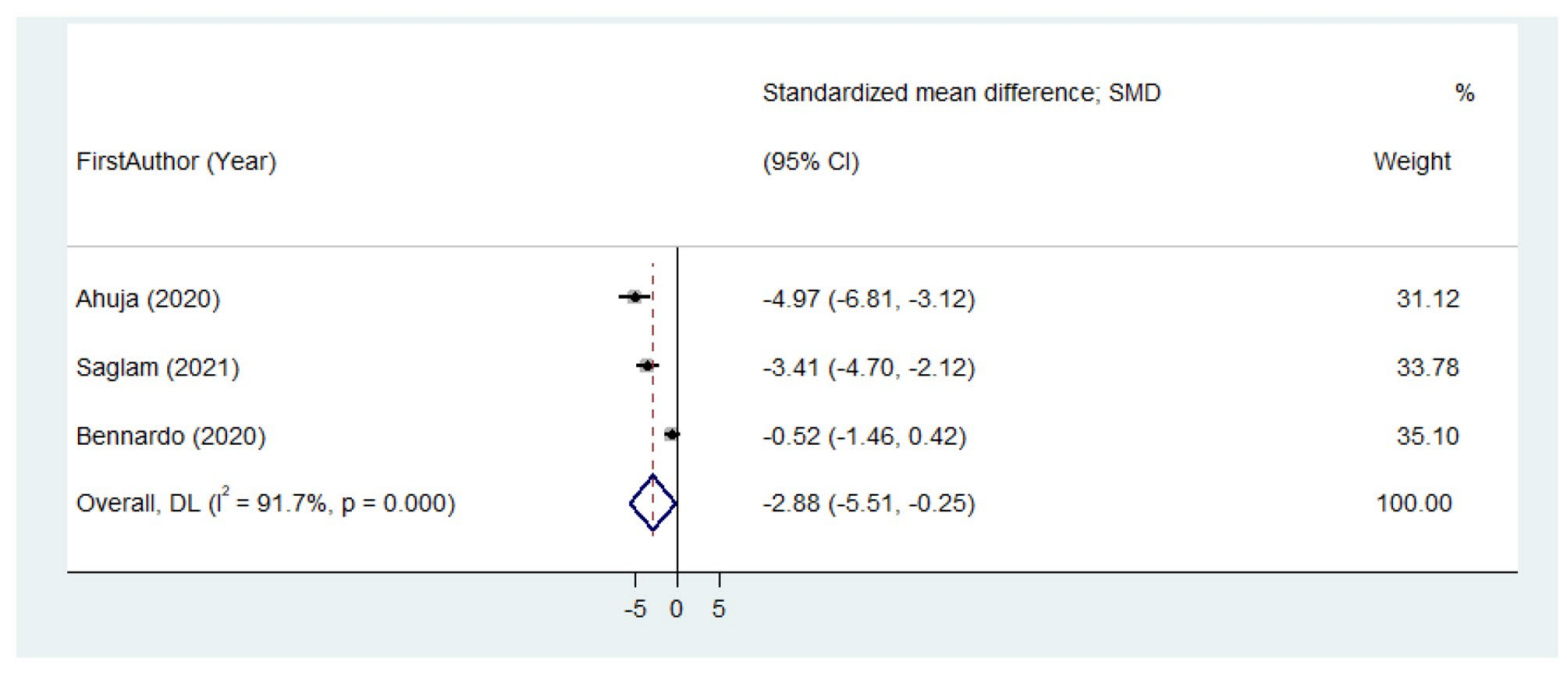
Meta-analysis of standardized mean difference of Thongprasom score

All of the six studies were included in the meta-analysis. Clinical parameters before the treatment and after the follow-up period were compared. There was no significant difference in the pooled estimate SMD of pain decline in patients receiving APCs in comparison with topical steroids (SMD = 0.17 (95% CI: -0.47 to 0.81); I^2^ = 63.6%) (Fig. 3). Meta-analysis showed that the SMD of Thongprasom score in patients receiving APCs was lower than the corticosteroid groups, with no significant effect size. (SMD= -2.88 (95% CI: -5.51 to -0.25); I^2^ = 91.7%) (Fig. 4). Since there were less than ten studies in each meta-analysis subgroup analysis and assessment of publication bias were not conducted.

## Discussion

This systematic review evaluated APCs as an alternative to topical steroids for managing symptomatic OLP. Lichen planus is an inflammatory disorder of the skin and mucous membranes with no known cause [32]. The currently available treatments only decrease the symptoms [33]. A variety of therapeutic options are used for the management of OLP, including corticosteroids, immunosuppressive agents (Cyclosporin, Azathioprine, and mycophenolate mofetil), and immunomodulatory agents (thalidomide and levamisole) [34]. 

Platelet concentrates (PCs), represented mainly by platelet-rich plasma (PRP) and platelet-rich fibrin (PRF), are autologous biological blood-derived products that may combine plasma/platelet-derived bioactive components, together with fibrin-forming protein able to create a natural three-dimensional scaffold. These products are safely used in clinical applications due to the autologous-derived source and the minimally invasive application procedure [35]. Autologous platelet concentrates have been used in medicine and dentistry for regenerative procedures and seem mainly to promote soft-tissue wound healing by delivering more than natural concentrations of autologous growth factors [36]. 

APCs contain growth factors and cytokines. The local release of growth factors and cytokines contained in platelet alpha granules accelerates tissue repair and promotes wound healing. This effect is boosted upon combination with the fibrinolytic system, which is crucial for complete regeneration [37]. 

The pathogenesis of OLP is influenced by various cellular mechanisms that are mediated by various cytokines. Tumor necrosis factor α, IL-1, and IL-4 play a significant role in disease progression [8]. PRP promotes the production of anti-inflammatory cytokines. These cytokines help the activated macrophages regulate the effect of pro-inflammatory cytokines. Anti-inflammatory cytokines regulate inflammation by interacting with soluble cytokine receptors and cytokine inhibitors [37]. Furthermore, Oxidative stress might have a role in the development of OLP [39]. It has been shown that PRP treatment can prevent oxidative damage by activating nuclear factor type 2, which, leads to increased signaling of antioxidant response elements [40]. 

Concerning the recurrence of the lesions and the treatment side effects, most of the studies reported no/mild symptoms of recurrence and no/mild side effects for either treatment modality. In the study of Ahuja et al., during follow-up for the next two months after treatment, the patients treated with PRP showed no or less recurrence, with only one patient out of 10 showing mild erythema and slight burning in the 15th week. In the corticosteroid group, three patients out of ten showed recurrences of the lesion during follow-up with increased pain and erythema compared to the 8th week. Also, there were mild side effects noted in two patients in the steroid group, but none of the patients treated with PRP reported any adverse effects [26]. In the split-mouth study of Al-Hallak et al., only two patients (16.7%) described mild symptoms of recurrence on both sides of the buccal mucosa [29]. In the study conducted by Hijazi et al., the remission score after three months of follow-up showed no significant difference between TA and PRP [28]. In the split-mouth study conducted by Saglam et al., no systemic side effects were reported for PRF or methylprednisolone acetate during the injections or the follow-up period [31]. In the study conducted by El Ghareeb et al., there was a significant increase in the frequency of side effects, especially pain, among patients who received PRP compared to those treated with steroids; this is in contrast with the other two studies that used PRP. This contrast may be due to lower injection intervals in this study and the dilution of TA with lidocaine as a local anesthetic. Also, there was a significant increase in recurrence rate among patients treated by PRP compared to TA; they suggested that this may be explained by the consumption of growth factors at the site of the lesion after a short period or by the immunosuppressive action of corticosteroids lasting for a long time [27]. 

The platelets’ function is not limited to hemostasis, but they have regenerative potential. PRP is a concentrated mixture of growth factors and cytokines that can influence inflammation, cell proliferation, stem cell migration, tissue repair, and angiogenesis [41]. Although the exact pathogenesis of OLP hasn’t been identified, it has been shown that many cytokines and inflammatory processes have an important role [42]. Therefore, it can be predictable that APCs might be useful in OLP’s management.

APCs may help patients with normal, impaired, and slower or incomplete healing by accelerating recovery. However, infection is one of the major contributors to delayed healing and tissue regeneration [43]. It has been suggested that using APCs as a drug delivery system, by combination with different molecules, such as antibiotics, can be useful [44]. Bennardo et al. reported that PRF could be loaded with antibiotics, and the drug is later released with antimicrobial effects [45]. Moreover, in vitro, research studied the effect of the addition of PRP to corticosteroids in chondrocytes and reported that the addition of PRP can significantly reduce the cytotoxic effects of corticosteroids [45]. 

Corticosteroids are the most commonly used medication for OLP due to their anti-inflammatory effects, nevertheless they are not definitive cures and only act in reducing the symptoms [47]. APCs however, could release various growth factors which endorse tissue repair, cell migration, angiogenesis, and tissue regeneration [48]. Additionally, APCs actively increase the proliferation of endothelial cells and fibroblasts [49]. Therefore it might be suggested that APCs could locally reverse the OLP lesions. The development of an effective three-dimensional fibrin scaffold following the administration of plasma rich in growth factors could facilitate healing, and guiding cell populations to their position and function [50]. More research is needed to evaluate the long term and probably definitive treatment effects of these preparations.

This review showed that platelet concentrates have the potential to alleviate the symptoms of OLP, have low side effects, and have a low rate of symptom recurrence. The results of treating OLP with APCs are comparable to topical steroids, and they have the advantage of lower side effects, such as oral candidiasis, which is seen with corticosteroids. Therefore, they can be suggested to be used, especially in patients who don’t respond well to topical steroids. Furthermore, future research is needed on using APCs as drug delivery systems for corticosteroids. Although there wasn’t enough information to compare the PRP with PRF, PRF may have a faster clinical response than PRP in managing OLP. Further studies are needed to compare these two materials.

This review had some limitations, such as the limited number of studies that have compared APCs and topical steroids, and as a result, the small size of the total sample, the heterogenicity of the outcomes, or the measurement scales of certain outcomes in different studies and the different time intervals of injections in the studies. Also, the follow-up times were different, which could affect the outcome results.

Within the limitations of our study, APCs could be effective in treating oral lichen planus and have comparable results with topical steroids. However, they have no superiority over topical steroids regarding the reduction of pain and clinical appearance. Furthermore, the higher expenses of APCs should be considered when choosing between these two treatment modalities. Future studies with larger sample sizes and longer follow-ups are recommended. Furthermore, it is suggested to conduct studies to reach a standard treatment protocol regarding the duration and intervals for using APCs in OLP patients.

## Conclusion

APCs were found to decrease the size of lesions, Thongprasom score, and pain in OLP patients; However, no significant differences were found between APCs and topical steroids. Thus, APCs could be considered as an alternative treatment to topical steroids. However, the results should be interpreted cautiously due to the high heterogenicity between the studies and a limited number of patients. Further well-designed prospective randomized clinical trials with large sample sizes and longer follow-ups are recommended.

### Electronic supplementary material

Below is the link to the electronic supplementary material.

Supplementary Material 1

## Data Availability

All data generated or analyzed during this study are included in this published article and its supplementary information files.
