# Supplementary material for: Comparison of autologous platelet concentrates and topical steroids on oral lichen planus: a systematic review and meta-analysis
Source: BMC Oral Health. 2024 Jun 8;24:674. doi: 10.1186/s12903-024-04443-y (PMC11162098; doi:10.1186/s12903-024-04443-y)
Supplement: Supplementary file 1 — Supplementary Material 1 [file 12903_2024_4443_MOESM1_ESM.docx]

| Database | Search terms | Articles found |
| --- | --- | --- |
| Embase | ('oral lichen planus'/exp OR 'lichen planus, oral' OR 'oral lichen planus' OR 'oral lichenoid reaction'/exp) AND ('platelet-rich fibrin'/exp OR 'l-prf' OR 'p-prf' OR 'leukocyte- and platelet-rich fibrin' OR 'leukocyte-prf' OR 'platelet-rich fibrin' OR 'pure prf' OR 'pure plaletet-rich fibrin' OR 'thrombocyte-rich fibrin' OR 'thrombocyte rich plasma'/exp OR 'platelet rich plasma' OR 'platelet-rich plasma' OR 'thrombocyte rich plasma') | 6 |
| PubMed | 1: ("oral lichen planus" OR "lichen planus, oral "[MeSH] OR "oral lichenoid reaction") AND ("platelet-rich-fibrin" OR "platelet-rich fibrin"[MeSH])  2: ("oral lichen planus" OR "lichen planus, oral "[MeSH] OR "oral lichenoid reaction") AND ("platelet-rich plasma "[MeSH] OR "platelet-rich- plasma")  3: (("oral lichen planus"[All Fields]) OR ("oral lichenoid lesions"[All Fields]) OR ("oral lichenoid reactions"[All Fields])) AND (("platelet rich plasma"[All Fields]) OR ("platelet rich fibrin"[All Fields])))  4: (oral lichen planus) AND (platelet rich plasma)  5: (oral lichen planus) AND (platelet rich fibrin)  6: ("Lichen Planus, Oral"[MeSH] or "oral lichen planus"[All Fields] or "olp"[All Fields] or "oral lichenoid lesion"[All Fields] or "oll"[All Fields]) AND ("platelet-rich plasma" [MeSH Terms] OR ("platelet-rich" [All Fields] AND "plasma" [All Fields]) OR "platelet-rich plasma" [All Fields] OR ("platelet" [All Fields] AND "rich" [All Fields] AND "plasma" [All Fields]) OR "platelet rich plasma" [All Fields])  7: ("Lichen Planus, Oral"[Mesh] or "oral lichen planus"[All Fields] or "olp"[All Fields] or "oral lichenoid lesion"[All Fields] or "oll"[All Fields]) AND ("platelet-rich fibrin" [MeSH Terms] OR ("platelet-rich" [All Fields] AND "fibrin" [All Fields]) OR "platelet-rich fibrin" [All Fields] OR ("platelet" [All Fields] AND "rich" [All Fields] AND "fibrin" [All Fields]) OR "platelet rich fibrin" [All Fields]) | 36 |
| Scopus | ( ALL ( "oral lichen planus" ) OR ALL ( "oral lichenoid reactions" ) OR ALL ( "oral lichenoid lesions" ) OR ALL ( "lichen planus oral" ) AND ALL ( "platelet-rich-plasma" ) OR ALL ( "platelet-rich-fibrin" ) OR ALL ( "platelet rich fibrin" ) OR ALL ( "platelet rich plasma" ) OR ALL ( "thrombocyte rich fibrin" ) OR ALL ( "thrombocyte rich plasma" ) ) | 130 |
| Web of Science | 1. (ALL=("oral lichen planus")) AND ALL=("platelet rich fibrin") 2. (ALL=("oral lichen planus")) AND ALL=("platelet-rich-fibrin") 3. (ALL=("oral lichenoid reaction")) AND ALL=("platelet rich fibrin") 4. (ALL=("oral lichenoid reaction")) AND ALL=("platelet-rich-fibrin") 5. (ALL=("oral lichen planus")) AND ALL=("platelet rich plasma") 6. (ALL=("oral lichen planus")) AND ALL=("platelet-rich-plasma") 7. (ALL=(" oral lichenoid reaction ")) AND ALL=("platelet rich fibrin") 8. (ALL=(" oral lichenoid reaction ")) AND ALL=("platelet-rich-fibrin") 9. (ALL=(oral lichen planus)) AND ALL=(platelet rich fibrin) 10. (ALL=(oral lichen planus)) AND ALL=(platelet rich plasma) 11. (ALL=("oral lichen planus")) AND ALL=(thrombocyte rich fibrin) 12. (ALL=("oral lichen planus")) AND ALL=(thrombocyte rich plasma) 13. (ALL=("oral lichenoid reaction")) AND ALL=(thrombocyte rich fibrin") 14. (ALL=("oral lichenoid reaction")) AND ALL=(" thrombocyte rich plasma") | 26 |
| Cochrane library | 1. "oral lichen planus" in All Text AND "platelet rich plasma" in All Text 2. "oral lichen planus" in All Text AND "platelet rich fibrin" in All Text | 11 |
